# Alveolar Abscess with Necrosis of the Maxilla

**Published:** 1871-10

**Authors:** H. L. Sage


### ARTICLE VII.

### *Alveolar Abscess with Necrosis of the Maxilla.*

By H. L. Sage.

October 29, 1870. Miss O. N., a young woman of Scotch and Irish parentage, bilio-lymphatic* temperament, called with the intention of having the second left superior bicuspid extracted, and replaced by an artificial substitute; also, the root of the first bicuspid.

On examination, a fistula was discovered over the root of the former; otherwise the gums presented as healthy an appearance as they usually do when thus affected, and the

---

*Though the bilious and lymphatic temperaments are said to be opposites yet they may exist together, one preponderating over or controlling the other.

tooth being of good quality, firm, and not badly decayed, the treatment of the abscess was advised and consented to.

An amalgam filling in the anterior approximal surface, which had been inserted by a neighboring dentist, about four years previously, was removed, and an opening found leading into the pulp cavity, which was empty.

The patient could not inform me, whether the pulp was destroyed previous to the insertion of the filling, or not. The trouble commenced to be *painfully* apparent, about two years after the tooth was filled, but was not noticed prior to that time. Either the pulp was dead at the time of filling, or was nearly or quite exposed.

The ultimate result, was alveolar abscess and necrosis of the maxilla.

The gums had recently swelled, but the swelling had subsided. The usual treatment was resorted to, and the abscess thoroughly cauterized *once*. This was done by cleaning out the pulp canal, and introducing a hypodermic syringe, loaded with a proper quantity of creosote into the root, and hermetically sealing the cavity about the point by filling it with a batter of plaster, which was allowed to harden, and then forcing the creosote through with the piston.

The medicine disappeared, but none of it came to the surface, through the fistula in the gum, as it was expected would be the case. The inference, therefore, was that the destruction of the alveolus by the abscess was considerable.

As the patient complained of a burning sensation, it may be, that some of the creosote found its way into the antrum.

The application was made November 2d. The patient afterwards called several times.

The gums became inflamed and livid, and the tooth very loose, and also the adjoining cuspid. There was very little if any pain. An endeavor to reduce the inflammation was unsuccessful.

It being feared by the appearance, that the antrum might become involved, the second bicuspid, together with the remnant of the root of the first, was extracted.

December 2d. Patient again called. On examination, much pus was found oozing from the sockets where the teeth had been extracted, and the cuspidatus was very loose. Extracted this with my fingers.

A large sequestrum of dead bone, embedded in the gums, but entirely detached, was removed. The piece extracted extended from the alveolus of the first molar anteriorly, to that of the lateral incisor posteriorly, and composed the sockets of the cuspidatus and of the first and second bicuspids, including a portion of the floor of the antrum, $\frac{1}{4}$ of an inch in diameter.

The length of the sequestrum, was $\frac{7}{8}$ of an inch: width, $\frac{5}{8}$; thickness, where not destroyed about $\frac{1}{4}$ of an inch. On the lingual portion, commencing $\frac{1}{4}$ of an inch above the inferior surface, it had been destoyed, leaving probably, one-half of its thickness.

The remaining teeth were firm. The opening into the antrum, over the root of the affected tooth, (second bicuspid) was at least one quarter of an inch in diameter.

The antrum was syringed with tepid water, and a weak solution of the chloride of zinc.

February 7, 1871. In about two months, the patient again made her appearance. There had been some discharge from the antrum, through the natural opening, of a light-colored, thin, and rather offensive fluid, which had entirely ceased about ten days previously—and the parts from which the sequestrum was taken, were looking quite healthy.

The opening in the floor of the antrum was closed, but the lips of the chasm, on the lower portion, though lapped together, were not entirely united. By scraping the edges the desired effect was produced.

The feautures suffered no deformity, and the loss was made good by the insertion of a rubber plate with teeth attached.

The cause of the necrosis is somewhat obscure.

Though no unusual inflammation of the gums was apparent when the case was first seen, or at least, not enough to excite observation, it is probable that the maxilla was in process of decay—dead or dying—only needing a little irritation, to produce acute inflammation, and cause speedy detachment of the sequestrum.

It is not reasonable to suppose that a single application of creosote would, of itself, produce inflammation, and necrosis, and that the process of inflammation, death and separation of the bone, would be accomplished in the space of one month. If the cause was not constitutional, or pro duced by a blow, of which there is no evidence, then in the absence of other known causes, it must be attributed to the abscess.

There is another idea in this connection: if the use of creosote and other caustic and irritating agents, or what would be *considered a judicious use*, would constitute a danger, by its occasionally finding its way into the antrum, and producing inflammation of the lining membrane, then this danger would arise in the treatment of alveolar abscess in connection with the bicuspids and molars, if their roots pierced the floor of that cavi y, or the destruction of bone was so extensive as to permit the entrance into it, of the agent employed for the cure of the abscess. Whether it would or would not, it affords a hint for the exercise of care in the treatment of these teeth, under such conditions.—*Dental Register.*

